# *Neurophotonics* book club: “The Secret of Secrets” by Dan Brown – a thought-provoking twist on a famous neuroscience controversy

**DOI:** 10.1117/1.NPh.13.2.020101

**Published:** 2026-06-24

**Authors:** Anna Devor, Gary Boas

**Affiliations:** a Boston University, Department of Biomedical Engineering, Boston, Massachusetts, United States; b Massachusetts General Hospital, Athinoula A. Martinos Center for Biomedical Imaging, Boston, Massachusetts, United States

## Abstract

This editorial explores the novel The Secret of Secrets by Dan Brown as a gateway to longstanding debates in neuroscience, particularly surrounding consciousness and free will. Highlighting the novels references to real neurophotonic technologies, it examines where scientific evidence ends and speculative fiction begins.

**Figure:**
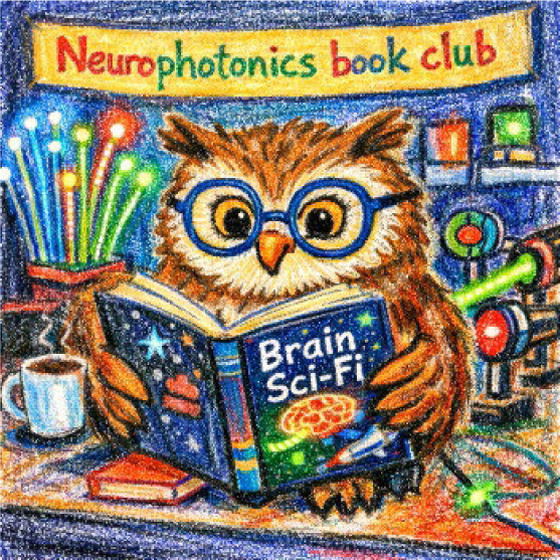


Dan Brown’s *The Secret of Secrets* picks up the story of everyone’s favorite symbologist: Harvard professor Robert Langdon, whom readers first met in Brown’s 2003 gazillion-seller *The Da Vinci Code*. Only this time, instead of unraveling a millennia-old mystery by deciphering symbols hidden in the paintings of Leonardo da Vinci, Langdon is caught up in a “race to harness the power of the human mind,” a race that involves a manuscript detailing a revolutionary theory of human consciousness and the shadowy forces seeking to suppress its publication.

*The Secret of Secrets* is a charming book and an honest-to-goodness page turner, but it’s also quite provocative in its way. Much like *The Da Vinci Code* sparked conversation and the occasional heated debate about the early history of Christianity, *The Secret of Secrets* raises challenging questions about the nature of consciousness.

And underpinning all of it is Brown’s remarkably well-researched depictions of neuroscience including multiple references to specific optical technologies for measurement and manipulation of brain activity. This is where Robert Langdon’s world, a world of kabbalistic legend and off-the-books government organizations jockeying for position in a struggle for global domination, intersects with the world of *Neurophotonics*’ readership.

*****

From the opening paragraphs of its prologue, in which a surprised neuroscientist, Brigita Gessner, finds herself floating above the city of Prague during an out-of-body experience, *The Secret of Secrets* walks a line between real science and speculative fiction.

Brown has clearly done his neuroscience homework. For example, one of the novel’s central plot points seems to have been inspired by a landmark study known today as simply “Libet’s experiment.” In 1983, Benjamin Libet, an American neuroscientist of Ukrainian descent, published a manuscript with a rather unwieldy title: “Time of Conscious Intention to Act in Relation to Onset of Cerebral Activity (Readiness-Potential): The Unconscious Initiation of a Freely Voluntary Act.” The work described how, during a human electroencephalography (EEG) study with a self-initiated voluntary movement (a finger tap), a wave of electrical activity — the “Readiness Potential” — started building in the motor cortex roughly half a second *before* the subject consciously decided to perform the tap. Sounds weird, right? Libet thought so, too.

The publication landed with a splash. In the years that followed, the basic result was reproduced by other investigators and vigorously debated by neuroscientists and philosophers alike. The big question was: If the brain activity associated with a decision occurs before we are conscious of making the decision, are we not in fact in control of our actions? And if not, do we bear a moral responsibility for the actions we take? Heady stuff.

It wasn’t until a quarter-century later, with a growing recognition of the “brain state” concept, that an alternative explanation appeared. The apparent answer to the longstanding mystery? That’s right, spontaneous fluctuations in neuronal activity.

Here’s how it works. Spontaneous fluctuations are happening all the time in the brain, and if we are told to make a voluntary movement without a temporal cue, “the precise moment at which the decision threshold is crossed leading to movement is largely determined by spontaneous subthreshold fluctuations in neuronal activity.” In other words, the tide of spontaneous neuronal activity rises and falls, and if we are free to initiate a movement at any time, we simply do it at high tide. We act when our “brain state” is favorable for taking an action, the stars are aligned, and we feel ready. So, we may be responsible for our actions after all.

Here is where *The Secret of Secrets* veers into the realm of speculative fiction, with … let’s call it an “original” reading of Libet’s experiment. In the story, an experiment similar to Libet’s shows a brain response occurs before the subject sees an image. Noeticist Katherine Solomon presents this as evidence of precognition, which she then uses to support her theory of nonlocal consciousness, in which the brain is viewed as a “receiver” tapped into a universal consciousness located outside the physical human body.

So, in the world of *The Secret of Secrets*, where flights of fancy are grounded enough in reality to give them a modicum of plausibility, a visual stimulus is substituted for a movement output, the brain is shown to predict the type of image even before it is randomly generated by a computer, *et voila*. Sure, the idea is maybe a bit preposterous, but it’s called science FICTION for a reason. If nothing else, we applaud the author for a well-sourced premise.

*****

Brown’s attention to detail doesn’t stop there. We also love the appearance of real-world neurophotonic technologies, and we were thrilled when Langdon and Solomon discovered functional near-infrared spectroscopy (fNIRS) while exploring a clandestine government facility:

*As they pushed deeper into the suite, they came upon a small, ride-on forklift with a massive crate in its tongs. Katherine crouched down to read the labels on the crate. “NIRS,” she said. “Near-infrared spectroscopy. Advanced real-time imaging.”*

*“In a medical facility?” Langdon associated NIRS with astronomy.*

*“Neuroscientists use it to analyze brain activity by assessing oxygen saturation.” Katherine stood, a look of concern in her eyes. “I don’t understand … Why would the CIA build a secret hospital under Folimanka Park?”*

Believe it or not, this isn’t the first time neurophotonic technologies have been implicated in sci-fi portrayals of precognition. In the 2002 film *Minority Report*, diffuse optical tomography was specifically named as a means to monitor the brain activity of “precogs,” people with psychic abilities who can perceive murders before they are committed.

Even two data points can suggest a trend. If Brown or any other writer would like to continue this trend, here are some neurophotonics technologies they can use.

First, we humbly suggest they consider hyperscanning. Hyperscanning measures inter-brain synchrony by simultaneously imaging the brain activity of two or more subjects interacting, but it could easily be adapted as a plot mechanism driving a spooky kind of action sequence involving multiple brains and unfolding across great distances. We also love the appearance of fluorescent artificial neurons and the ‘fire together wire together’ neuroscience principle. Of course, fluorescence relies on the absorption of light from an external source, and we found no mention of miniaturized lasers or the like implanted in the brain of Brigita Gessner’s lab assistant Sasha Vesna. As an alternative, we respectfully suggest a virally delivered gene for infrared bioluminescence that can be activated by ingesting a substrate (chemical) that crosses the blood–brain barrier. No surgery required!

Anyway, just a few thoughts. You’re welcome, literary world.

*****

One of the major themes of the book is the tension between weaponizing brain science — and the brain itself — and forging a path toward a kind of enlightenment guided by new understandings of consciousness. In some ways (though not, of course, in all ways; the “weaponizing the brain” part doesn’t apply here), this tension echoes a tension in real-world approaches to understanding consciousness.

Of these approaches, the more prominent view in contemporary neuroscience is the materialist position, which holds that consciousness is entirely the result of physical brain processes — that subjectively felt experience can be reduced to neural firing patterns, electrochemical dynamics, or even quantum mechanical processes and that, eventually, we will have read and write technologies powerful enough to interface with these processes.

Philosophers, religious thinkers and parapsychologists push back against this idea, arguing that there must be something more. Not even the most detailed map of the brain, they say, would fully explain how and why we have consciousness. (In *The Secret of Secrets*, Solomon was trained as a neuroscientist with materialist views but ultimately adopted the thinking of noetics.)

In modern times, scientists have often dismissed the “something more” idea, not least because science is powered by measurable, reproducible evidence, and intangibles like “consciousness as a fundamental feature of the universe” don’t sit comfortably within that framework. But recent decades have seen a sort of chipping away at this bias, with the emergence of a wide range of theories of consciousness—some of which include a metaphysical component – and with prominent figures like Francis Collins describing truth, science, faith, and trust as the four pillars of wisdom.

We believe that brushing away the metaphysical simply because it resists measurement would be a mistake. The history of knowledge, after all, is often the history of insights coming from well outside the boundaries of conventional wisdom and unchallenged premise. In the end, keeping an open mind to ideas from philosophy, religion and other traditions doesn’t have to mean we are in conflict with scientific doctrine.

In any event, with further advances in neurotechnologies, such claims may one day be testable. Then again, maybe they won’t. Another in the history of ideas seeking to reconcile scientific and religious thought is the Watchmaker Analogy, which posits that the laws of nature didn’t come about by accident, but rather were designed and set in motion by some greater, unknowable power. As an example: Remember the rising and ebbing tides of spontaneous neuronal activity that finally explained the results of Libet’s experiment? Even today, we don’t really understand what controls these tides. And we may never understand. Perhaps, as the saying goes, “God only knows.”

